# Seeking Support: The Voice of Young Men Who Have Experienced Sexual Harm During Their Life Course

**DOI:** 10.1177/08862605241308297

**Published:** 2024-12-27

**Authors:** Tess Patterson, Linda Hobbs, Gareth J. Treharne, Louise Dixon, Alannah Corson Keogh, Melanie Beres

**Affiliations:** 1 University of Otago, Dunedin, New Zealand; 2 North-West University, Potchefstroom, South Africa; 3 Glasgow Caledonian University, UK

**Keywords:** sexual abuse of men, sexual assault of men, male survivors of sexual abuse, sexual harm of men

## Abstract

The sexual abuse and assault of boys and men is not uncommon, and seeking support is useful in reducing negative outcomes. However, male survivors are less likely than women to seek support. Gendered norms and myths persist with several gender-specific barriers to seeking support existing for men. The present study is guided by three research questions: (1) What are men’s support needs in relation to their experience of sexual harm? (2) What are men’s experiences of seeking support in relation to their experience of sexual harm? (3) What are the barriers that hinder men from seeking support? Interviews were conducted with 14 men (19–37 years old) attending one of two universities in New Zealand, who had experienced sexual harm. The interviews included a discussion of the men’s experiences of seeking support and were conducted as part of two broader projects. The young men experienced a complex and multifaceted journey in seeking support and described a range of informal and formal avenues that they had reached out to. Three prominent needs were highlighted: (1) increased awareness of male survivors, (2) a need for clarity around formal support services, and (3) a need for a diverse range of support modalities. The men described a variety of barriers encountered, including individual-level barriers such as difficulties in recognizing their experiences as sexual harm, a reluctance to acknowledge a need for support, preconceived notions about what seeking help might be like, as well as several overarching social and contextual influences such as living situations, cultural norms, religious beliefs, and family dynamics. These findings underscore the need for a holistic approach to supporting male survivors that addresses gender norms and myths about male survivors, acknowledges the diversity among male survivors, and addresses both individual-level and broader systemic barriers to support seeking by young men who have experienced sexual harm.

## Introduction

Estimated prevalence rates indicate that as many as 3% to 17% of men experience childhood sexual abuse (Barth et al., 2013), and 3.8% to 15.6% experience adult sexual assault (Peterson et al, 2011). In Aotearoa New Zealand (NZ), it has been reported that 12% of men have experienced one or more incidents of sexual harm (including any type of forced or coerced sexual contact, assault, harassment, or behavior that happens without consent) during their lifetime (compared to 34% of women) (NZ Crimes and Victims Survey; Ministry of Justice, 2020). More specifically, prevalence rates suggest that between 3.2% and 28.7% of university-aged men have experienced sexual harm while a student (Eisenberg et al., 2021; Jouriles et al., 2022; Luetke et al., 2021; Patterson et al., 2022). Within the NZ context, Beres et al.’s (2020) study found that 13.2% of NZ university student men reported experiencing sexual victimization while at university.

Like female survivors, male survivors also suffer significant negative sequela following harmful sexual abuse (Petersson & Plantin, 2019), with adverse outcomes documented for both men and women in terms of mental health (Burch & Zepeda, 2023; Zarchev et al., 2022), physical health (Maniglio, 2009), and relational difficulties (Easton, 2012). Male survivors may also be at an increased risk, compared to female survivors, of experiencing post-traumatic stress disorder (PTSD) (Burch & Zepeda, 2023; Easton, 2012), suicidal ideation or suicidality (Easton et al., 2013; Nicholas et al., 2022), sexuality issues (O’Leary et al., 2017), and maladaptive coping behaviors such as alcohol or substance misuse, aggression and engagement in risk-taking behaviors (Burch & Zepeda, 2023; Easton, 2012; Rice et al., 2022).

It is well recognized that current prevalence rates likely underestimate actual rates of sexual harm experienced by males as many cases of sexual abuse or sexual violence, including those experienced as a university student, are never officially reported (Jouriles et al., 2022; Luetke et al., 2021).

While understanding the prevalence rates of those experiencing sexual harm is useful, more recently researchers and service providers have suggested a shift away from a sole focus on the number of men who have experienced sexual harm toward research that provides a better understanding of men’s experiences and support needs (Dixon et al., 2023; Wingender & Olesen, 2023). Seeking support, intervention, and treatment is shown to be helpful in reducing the long-term negative effects of sexual harm (Oosterbaan et al., 2019; Rapsey et al., 2020). Delayed disclosure or non-support from others may worsen detrimental outcomes (Burch & Zepeda, 2023). It is therefore important to more fully understand the barriers that hinder disclosure and support seeking for male survivors (Easton et al., 2014; Machado et al., 2016).

There is, however, a paucity of research examining the support and treatment needs (Burch & Zepeda, 2023; O’Gorman et al., 2024) or support-seeking behaviors for men who have experienced sexual harm, especially university students (Beres et al., 2020; Carlisle & Schmitz, 2022; Dixon et al., 2023; Wingender & Olesen, 2023). The limited research conducted to date indicates that support seeking by males is less likely to occur compared to females. For example, a U.S. survey indicated that university students perceived there were barriers that hindered men from disclosing sexual harm and seeking help (Allen et al., 2015). In a study of NZ university students, Patterson et al. (2022) found that male students are less likely than female students to seek support and men who do seek support are less likely than women to seek it from formal services.

Research has identified some barriers that may explain these low rates of male help-seeking for experiences of sexual harm, including some that are gender-specific (Dixon et al., 2023; Lowe & Belfour, 2015; Murphy-Oikonen & Egan, 2022; Rapsey et al., 2020). Identified barriers include fear of negative consequences, shame, and self-blame (Burch & Zepeda, 2023; Dixon et al., 2022; Pijlman et al., 2023), masculine norms, difficulty understanding, and labeling their experiences as abusive (Artime et al., 2014; Weare et al., 2024), and a lack of awareness about relevant support (Weare et al., 2024).

One gender-specific barrier to seeking support for sexual harm that has been discussed is that of hegemonic masculinity and how a man is typically defined by social constructs of society and culture (Burch & Zepeda, 2023; Weare et al., 2024). In NZ, persistent masculine norms suggest the way in which boys and men are expected to think and act. This includes being self-reliant and demonstrating strength, toughness, and power over others (Burch & Zepeda, 2023). Reaching out for support is often contrary to these masculine norms (Andersen, 2013). As a result of societal expectations and gender norms that dictate what it means to be masculine, boys’ and men’s masculinity can often be reinforced through shame. From a young age, boys are socialized to “toughen-up” and suppress emotions, vulnerability, and expressions of pain, as these qualities are often associated with weakness and femininity (Mejia, 2005). This suppression can lead to a deep sense of shame and silence when boys and men experience trauma or challenges, including instances of sexual harm (Easton et al., 2014). Often the experience of sexual harm conflicts with or undermines these masculine norms, values, and identities (Carlisle & Schmitz, 2022; Kia-Keating et al., 2005; O’Gorman et al., 2024). These conflicting messages may not only result in gender-specific challenges (e.g., higher rates of PTSD or suicide ideation compared to women) but also in gender-specific barriers that hinder men from seeking help or support (Andersen, 2013; Monk-Turner & Light, 2010).

Many male survivors fear judgment, disbelief, or ridicule if they disclose their experiences (Weare et al., 2024), as there is a prevailing stigma surrounding male victimhood. Moreover, societal expectations dictate that men should be strong, stoic, and able to protect themselves, making it difficult for them to admit vulnerability or seek help (Andersen, 2013). In addition, the powerlessness experienced during sexual abuse or sexual violence may be replayed during interactions with healthcare or judicial systems (Gallo-Silver et al., 2014; Machado et al., 2016). Research focused on female survivors suggests that many also experience secondary victimization during interactions with healthcare or judicial systems where they may encounter unresponsive interactions or may be required to replay their negative experiences numerous times (Campbell & Raja, 1999). This is likely the case also for male survivors (Jackson et al., 2017; Thomas & Kopel, 2023), with those who sought help endorsing greater distrust of support providers (Pijlman et al., 2023) than non-help-seekers.

Furthermore, persistent myths surrounding male sexual harm perpetuate harmful misconceptions and alongside traditional masculine norms hinder both recognition and support for male survivors (DeJong et al., 2020; Turchik & Edwards, 2012). For example, one prevalent myth suggests that men cannot be victims of sexual harm, as it is often perceived as a crime primarily affecting women (Depraetere et al., 2020; Easton et al., 2014; Hammond et al., 2017). Similarly, male survivors often experience feelings of helplessness, shame, and silence because of the myth that sexual abuse implies weakness or emasculation (Kia-Keating et al., 2005). Another persistent myth is that male survivors are automatically gay. As a result, male survivors may struggle with feelings of confusion and shame, fearing judgment and ridicule from others (Widanaralalage et al., 2022). Additionally, there is a myth that male survivors are more likely to become perpetrators themselves, despite evidence to the contrary (Burton et al., 2002; Kia-Keating et al., 2005; Salter et al., 2003). These persistent myths not only invalidate the experiences of male survivors but also discourage them from seeking help and speaking out about their trauma, perpetuating a cycle of silence and suffering.

Persistent societal masculine norms and myths result in difficulty for men contextualizing their experience as “abusive” and reject the idea that males can be “victims” (Burch & Zepeda, 2023; Carlisle & Schmitz, 2023; Depraetere et al., 2020). Compared to women, men are less likely to self-identify as victims or recognize their experience as abusive (Andersen, 2013; Carlisle & Schmitz, 2022; Depraetere et al., 2020; Easton et al., 2014; Machado et al., 2016) or that their experience was not normative (Willis et al., 2014).

Overall, past research has indicated that men who have experienced sexual harm may encounter a great number of barriers when reaching out for support, many that stem from gendered norms and persistent myths around male sexual harm (Rapsey et al., 2020). There is, however, a paucity of research that examines the support needs, experiences and barriers encountered when young men who have experienced sexual harm reach out for support (Wingender & Olesen, 2023). A better understanding of these needs, experiences, and barriers encountered can better inform service providers and future research in ways to better support men who have experienced sexual harm.

The present study aims to explore the support needs and experiences of seeking support from a group of university student men. This study is guided by three research questions: (1) What are men’s support needs in relation to their experience of sexual harm? (2) What are men’s experiences of seeking support in relation to their experience of sexual harm? and (3) What are the barriers that hinder men from seeking support?

## Method

### Study Design

A qualitative approach was chosen as the most appropriate method for exploring the complex dynamics inherent in sexual harm research (Gibson & Morgan, 2013). The University of Otago Ethics Committee (Institutional reference 19/065) and the Victoria University of Wellington’s Human Ethics Committee (project number 28954) gave approval for the two studies that were combined in this analysis.

### Sample Characteristics

Participants selected for this analysis who were attending university and had been interviewed as part of one of two broader projects: (1) the Campus Climate Survey (CCS) 2019 (Beres et al., 2020), and (2) the Male Survivors of Sexual Violence and Abuse (MSSVA) project in Wellington and Otago (Dixon et al., 2023). All participants had completed an in-depth semi-structured interview that included a discussion of their experience of sexual harm and of seeking support. Those who participated in the CCS had experienced sexual harm since enrollment at university and those who participated in the MSSVA study had experienced sexual harm as a child and/or as an adult. Prior to participation in the semi-structured interview, information was given that emphasized that participation was entirely voluntary, and individuals provided written consent.

A total of 14 (3 from the CCS; 11 from the MSSVA study) interviews with young adults who identified as men and had experienced sexual harm were included. Interviews were with men aged between 19 and 37 years (*M*_age_ = 26 years; SE = 1.38), 11 of the men identified as New Zealand European, 2 as New Zealand Māori, and 1 as “other” ethnicity. Four men reported they had experienced sexual harm in childhood, six reported they had experienced harm during adolescence and four during adulthood (some experienced harm during more than one phase of their life; data not available for three men); two reported they had experienced one harmful sexual event, three reported they had experienced more than one event by the same person, and six reported experiencing more than one event by different people (data not available for three men); six men reported the person who engaged in the harmful behavior was male, two reported it was a female, and three reported experiencing harm from both a male and female (data not available for three men); and three men reported an intra-familiar connection with the person who sexually harmed them (data not available for three men).

### Analytic Approach

NVivo, a qualitative data analysis software package, was used to assist with the identification and classification of themes. Reflexive thematic analysis (RTA) was employed to explore participants’ experiences. RTA is a bottom-up thematic analysis method and thus is not used to test hypotheses but to understand the meaning that individuals in a particular context make from their experience (Braun & Clarke, 2022; Smith & Osborn, 2015). It is an iterative process that involves multiple readings and immersion in the text to confirm that the themes and interpretations are supported by the data. The process of thematic analysis is not a discrete step but continues throughout the analysis and write-up phases. RTA acknowledges that a participant’s account during the interview is an attempt to make sense of their personal world and social experience (Larkin et al., 2006). It is also acknowledged that the interviewer interprets the participant’s account through the lens of their own attitudes and experiences (Smith & Osborn, 2015). Thematic analysis and identification of themes were guided by the three research questions. Differences were discussed by the team to formulate a consensus on themes.

## Results

### Question 1. What Were the Men’s Support Needs in Relation to Their Experience of Sexual Harm?

**Figure 1.**
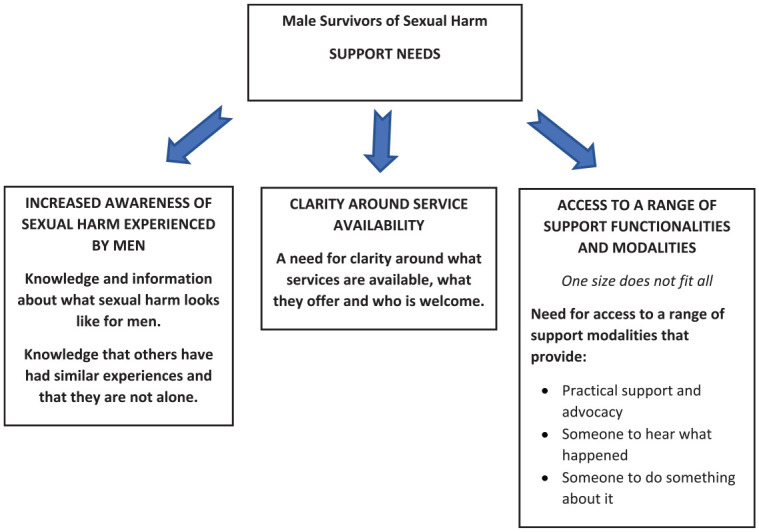
Support needs of men who have experienced sexual harm.

Throughout the interviews, the men provided insights into their support needs and how well their needs were met. We identified three themes that illustrate the men’s descriptions of their support needs: (a) increased awareness of sexual harm experienced by men; (b) a need for clarity around what services are available, what they offer, and who is welcome; and (c) a need for a range of modalities of support. Figure 1 provides a summary of these themes. Each theme is illustrated with quotes from the men.

#### Increased Awareness of Sexual Harm Experienced by Men

The men talked about a need to have more information and knowledge about what sexual harm looks like for men.

“So, I would think even just seeing words or hearing a sound, that someone’s speaking these words, ‘This happens to men. Men get raped. Men get abused,’ and even just normalising that would promote or at least contribute towards promoting a speak up culture within men that have been affected and are affected by it.” [Participant 21]

They suggested that this may help them realize that they are not the only person that this has happened to, that they are not alone and it is ok to ask for help or to tell someone about their experience.

“. . . also the visibility thing of just realising it’s normal would have helped a lot as well. Just having any sort of something I can relate it to that didn’t make me feel so isolated . . . that would make it a lot easier for me to talk about it.” [Participant 30]

#### Clarity Around Service Availability, What They Offer and Who Is Welcome

The men talked about a need for services to be clear about what they offer and whether they are welcoming to men who have experienced sexual harm.

“Be explicit about it. If you are welcoming to male victims of sexual violence, say so.” [Participant 1]

“so I feel weird about groups that cater for women and other gender minorities because it’s pretty much they’re like for everyone except for cis guys. . . . and I don’t really feel like I fit there.” [Participant 27]

Many voiced a hesitancy to contact a service with a fear that the service may not cater for men who have been sexually harmed.

“if I weren’t able to ask someone questions about the structure, about the content, about how it goes for the session; I just don’t think I would have . . . been able to book a session myself . . . it’s just kind of that’s what it was for me, was being able to prepare myself, by knowing what to expect.” [Participant 10]

#### Access to a Range of Support Functionalities and Modalities

Throughout the interviews, the men expressed a wide range of needs, these included needing support to move out of a harmful living situation, support dealing with legal systems, and needing someone to talk to about their experience. The men talked about a need for a number of support functionalities to match these needs, such as those that provide practical support and advocacy (e.g., help with moving out of a harmful living situation or dealing with legal systems), those that are there to listen and to hear about their experience (e.g., support groups and talking therapies) and those that are there to do something about the situation (e.g., the Police). The men also suggested that a single service format may not suit all and that there is a need for a range of formats that include talking therapies (with a psychologist/counselor), anonymous helplines, and support groups.

“. . . access to a range of modalities, I think.” [Participant 1]

“I do think I would be more inclined to go to some kind of service if there was maybe like an anonymous helpline, or like a call thing where I don’t have a face, and I don’t have a case manager or whatever. . . . flexible options of going when you feel like it and was free.” [Participant 30]

“I know somebody who went to the [name of support service for women] and they help organise counselling and I think they do legal fees as well and they can help with furniture and stuff. I feel like that would be helpful if there was a thing for guys.” [Participant 27]

### Question 2. What Were the Men’s Experiences of Seeking Support in Relation to Their Experience of Sexual Harm?

**Figure 2.**
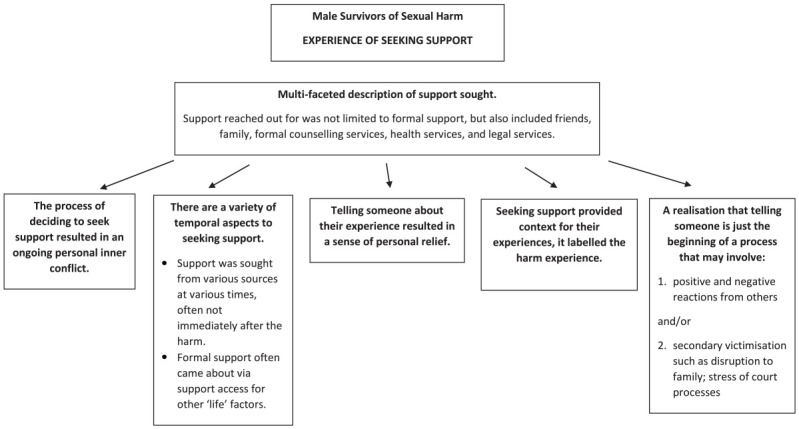
Experiences of seeking support for sexual harm.

Across the interviews, the men described a range of people and services that they had reached out to for support. This was not limited to formal support services; support avenues were talked about as multifaceted, including friends, family, formal supports such as counseling and health services, and legal reporting processes such as to the Police. We identified five themes that describe the men’s experiences of seeking support: (a) the process of deciding to seek support resulted in an ongoing personal inner conflict; (b) there are a variety of temporal aspects to seeking support; (c) telling someone about their experience resulted in sense of personal relief; (d) seeking support provided context for their experiences; and (e) a realization that telling someone is just the beginning of a process. Figure 2 provides a summary of these themes. Each theme is illustrated with quotes from the men.

#### The Process of Deciding to Seek Support Resulted in an Ongoing Personal Inner Conflict

Throughout the interviews, men voiced an ongoing inner conflict as to whether they wished to seek support or not.

“It (seeking support) was intense, and I didn’t want to do it, but I wanted to do it because it was like, well if we can work out how damaged I am we can work out how much help I need.” [Participant 27]

While acknowledging that seeking support was helpful or useful, some of the men expressed a want to get through their distress on their own without the help of others, whereas other men suggested that they didn’t want to think about what had happened and that seeking support would force them to think about it.

“Although there was support there; I didn’t really take it. I didn’t want it. I pushed it away. There was a part of me that thought I could get through it by myself. I didn’t want anyone else’s help.” [Participant 36]

#### There Are a Variety of Temporal Aspects to Seeking Support

Men reported that they sought support not only from a variety of sources (e.g., peers, parents, formal health services) but also at various time points after the harm, often not immediately.

“it was ongoing I think for a couple of years before I told my family and then we went to the police about it.” [Participant 1]

“Probably about in three or six months is where I place it, when I talked about it.” [Participant 10]

The men described that support from formal avenues (e.g., counseling) that addressed their experiences of sexual harm often came about when they accessed support for other “life” issues separate from the sexual harm.

“It just came up; yeah. It wasn’t like I was there to talk about the sexual abuse. It was just part of my up-bringing, and it just came up. What I was initially there to talk about was my historical injuries that I had suffered from a car accident.” [Participant 35]

#### Telling Someone About Their Experience Resulted in a Sense of Personal Relief

Several of the men expressed a sense of relief in telling someone about the sexual harm they had experienced. This relief was often talked about in the context of fear of telling someone or of the weight of having carried a secret for some time.

“I remember feeling quite relieved to have told someone . . . there was real fear about getting the words out, but it felt good to not be carrying this secret.” [Participant 1]

“It was nice for it just not to be this dark secret that I had anymore.” [Participant 6]

#### Seeking Support Provided Context for Their Experiences, It Labeled the Harm Experience

The men reported that seeking support and telling someone about their experience placed the experience in context and provided the experience with a concrete label. Placing their experience in context and labeling it was a positive aspect of seeking support.

“it (seeking support) has helped to contextualise what happened to me. I think it has been good in terms of it’s very easy to become defined by a traumatic event or traumatic events and seeing those memories in a larger context.” [Participant 1]

The men expressed that talking about their experience may help shift self-blame for the harm or help survivors define themselves in ways that are not solely based on the traumatic events that they have experienced.

“it was actually one of the nurses at [university healthcare service], that kind of said like ‘yeah you were sexually assaulted’ um which I, yeah I hadn’t thought of it like that before then. . . . it did help me stop blaming myself for what happened.” [Participant 4]

#### A Realization That Telling Someone Is Just the Beginning of a Process

The men also described that seeking support or telling someone about their experiences often was the beginning of a process and that the process may extend to other experiences beyond that directly related to the sexual harm event. Some of the men referred to secondary victimization where the experience of seeking support was also traumatic and induced its own long-term impacts. The men also talked about the disempowerment and re-traumatization they experienced throughout legal processes.

“I wanna call it um secondary victimisation where for me that was like part of the response for complaining, were more traumatizing than the initial event and has had more lasting impacts I would say.” [Participant 26]

“I think I felt very disempowered. I did not feel that I had agency over the experience. It felt very impersonal, and it compounded the trauma because being asked to describe in excruciating detail the abuse that I had suffered in a courtroom.” [Participant 1]

Some described disruption to their family that was brought about by disclosure of their harm and by ongoing legal court processes, which for some included a complete breakdown of family dynamics and relationships.

“My younger sister still I don’t think has really forgiven me (emphasis on me) for what happened. As far as she was concerned there was nice Uncle [name] who she saw every day and suddenly she wasn’t allowed to see him anymore and everything went very strange.” [Participant 1]

In addition to describing the impact of the negative reactions to their seeking of support the men also described positive reactions they received from seeking support.

“I find it very easy to talk to her because of those similarities in terms of our world view, and her, I guess empathy and compassion.” [Participant 10]

### Question 3. What Are the Barriers Encountered by the Men That Hindered Reaching Out for Support?

**Figure 3.**
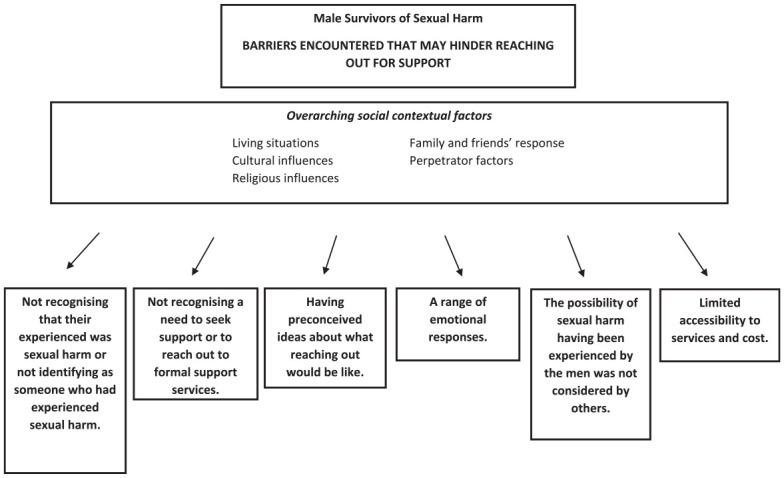
Barriers encountered to seeking support for sexual harm.

Throughout each interview, the men described a wide range of barriers that had held them back from seeking support for their experiences of sexual harm. It is pertinent to recall that the men described a wide range of people and services that they reached out to for support; similarly, they talked about a wide range of barriers that often related specifically to a certain type of support (e.g., peers, family, Police, and formal counseling). We identified six themes that describe the barriers to seeking support encountered by the men: (a) not recognizing that their experience was sexual harm; (b) not recognizing a need to seek support or to reach out to formal support services; (c) having preconceived ideas about what reaching out would be like; (d) a range of emotional responses; (e) the possibility of sexual harm having been experienced by the men was not considered by others; and (f) limited accessibility to services and cost. Additionally, we describe an overarching theme that includes a number of social contextual influences that the men also talked about in terms of barriers to seeking support. The men talked about how these social influences interacted with each of the six themes. Figure 3 provides a summary of these themes. Each theme is illustrated with quotes from the men.

### Not Recognizing That Their Experience Was Sexual Harm or Not Identifying as Someone Who Had Experienced Sexual Harm

When discussing barriers to seeking support, the men talked about not recognizing that their experience was sexual harm and underplaying the experience as something that wasn’t “major” and didn’t require reporting or reaching out for support.

“I wasn’t sure whether what I’d experienced counted as sexual assault.” [Participant 11]

“Early on I didn’t really think it did happen to men so that’s probably why I never sought help for it, I was uncomfortable by it, but I didn’t think it was bad in anyway.” [Participant 6]

“. . . also I wasn’t even sure if it was a thing or not. I was trying to work out if I was just being dramatic or if it was actually not okay.” [Participant 27]

As part of this men also talked about not having the language or knowledge to talk about their experiences as a barrier to seeking support.

“I think men need to know that it happens to them and it’s just as valid as for women. I think that’s the biggest deterrent is we just don’t know that what is going on is an offence and we just need to deal with it.” [Participant 6]

“just really not having the vocabulary . . . to be able to process this trauma.” [Participant 1]

“I was 14 by the time I realised that I’d been raped. I knew exactly what had gone on, but when I was 14 and I heard the word ‘rape’ for the first time, when someone told me what it was, . . . I went years without realising what a word was because I was so sheltered, and even though it had been happening to me every week for years. So, even just in terms of language, just is important for education.” [Participant 21]

#### Not Recognizing a Need to Seek Support or to Reach Out to Formal Support Services

The men talked about not recognizing that they needed to seek support or that they felt that they already had family support or the skills to deal with it or could figure it out themselves and didn’t need to reach out to formal support services.

“It’s always been in me a sense that I can figure it out by myself. And I think that’s a large part of what has prevented me [seeking support].” [Participant 16]

#### Having Preconceived Ideas About What Reaching Out for Support or Support Services Would Be Like

The men described preconceived ideas that they held about reaching out for support. They described a belief that formally reporting their harm would be unlikely to be taken seriously or unlikely to result in any further action being taken against the perpetrator so therefore was not something they wanted to pursue.

“There’s no evidence to begin with, so I don’t see it going further. And from what I’ve understood is it’s not really taken seriously even for women often, so the police force is quite a hyper masculine thing, so if you go reporting sexual assault as a man, is it going to be even taken seriously at all? Are they going to take the piss out of you, something like that?” [Participant 6]

They also described that a lack of understanding of the process for formally reporting a sexually harmful experience held them back from reporting. The men also described a hesitancy to seek counseling support that was often based on previous negative experiences that they had had with similar services.

“that experience of about a year or so when I was seeing that counsellor did nothing for me, so I was basing every other service, mental health service, I was basing it thinking it’s going to be the same as that. So, I think that actually if that was the only reason why I didn’t actually say, ‘Well yeah, actually, I will see someone.’” [Participant 21]

#### A Range of Emotional Responses Can Be a Barrier

Men talked about a wide range of emotional responses that hindered them in seeking support. The men talked about feelings of shame, embarrassment, and their fear of not being believed, of being blamed, of not being seen as masculine, and fear of possible disruption to their current life.

“Shame. Embarrassment. Definitely embarrassment. A feeling that no one would understand or relate.” [Participant 21]

“I think the biggest thing if I speak for myself was the fear of being scared; of being judged; of what people would say; of not believing you; of thinking you’re someone different.” [Participant 36]

“It [to tell someone] felt like a lot of pressure. It felt like too much of a cost to tell someone about it and the cost being that I would be blamed rather than actually anybody listening to me.” [Participant 21]

“Although that abuse was going on I still had a pretty good life from what I was having beforehand. I didn’t want to lose that. You’d lose all of this if you were to tell anyone so I didn’t tell anyone.” [Participant 36]

Men also talked about being hesitant to reach out for support because of the stigma associated with being labeled as a man who had experienced sexual harm, a desire to not appear weak (in needing help or as a result of experiencing sexual harm), and a need to protect their pride and mana.

“Like I said I had a reputation. I had pride. I had all this mana all for the wrong reasons but I still chose to hide that; mask it and just bury it so that; if I buried it and masked it; hid it away from everyone; no one would know. I wouldn’t have to speak about it. I’ll be the big macho man.” [Participant 35]

“I just don’t want people to know even if they’re in a place to help me, and even if it’s my therapist. I just don’t want these sort of labels attached to me . . . I just don’t want people to know that about me really, because I don’t see it as part of my identity. I don’t want people to associate me with that.” [Participant 30]

“It’s like booking into see a counsellor makes me feels like I’ve failed to keep my own emotions in check, and I’m seeking help, because seeking help is a weakness” [Participant 10]

#### The Possibility of Sexual Harm Having Been Experienced by the Men Was Not Considered by Others

Men talked about instances in their life where they felt that had they been directly asked about sexual harm in relation to their current situation and behavior (e.g., in relation to not attending school and drinking to excess as an adolescent) they may have talked about it earlier.

“. . . had anyone questioned why I wasn’t attending school, what was going on in my life here that had made me just go off the rails and never be at school, always be drunk, had an adult come in at that time and sat me down and tried to get to the bottom of what was going on, something there would have been great. But I was just seen as just another youth that was off the rails and disregarded.” [Participant 6]

“No, they didn’t [ask me]. . . . Maybe if they’d asked me straight, as in, ‘Are you actually bringing up some form of abuse from the past?’ If they’d asked me directly, just knowing how I’m like, I probably might have said. But they were just hitting all the wrong spots.” [Participant 21]

#### Limited Accessibility and Cost of Formal Support Services

In relation to seeking support from formal support services the men talked about limited accessibility to relevant services for men and the often-prohibitive expense of accessing formal support services.

“also there not really being support out there for guys, there’s the Women’s Refuge who help people with lots of side of things, and lots of things, but there isn’t a Men’s Refuge.” [Participant 27]

“The psychologists or psychiatrists are far too expensive. I would never be able to afford any long-term treatment there, or even short-term.” [Participant 6]

“I did benefit from counselling in high school and um they do have counsellors at Student Health that I’ve been offered by my GP’s a couple of times um but they’re actually not that easy to access.” [Participant 9]

### Overarching Social Contextual Factors: Social Circumstances That Interacted with Each of the Identified Themes

In addition to the six themes discussed above, the men also talked about a number of overarching social and contextual factors that hindered them from seeking support. The men talked about how these overarching social and contextual factors influenced and interacted with each of the themes outlined above. The men talked about their living situation (e.g., debt and lack of financial freedom and feeling they had no means to leave an abusive situation), cultural influences (e.g., negative peer group influences that reinforced feelings of shame and weakness around sexual harm), religious influences (e.g., lack of acceptance of sexuality and transgender men by the church community), family and friends beliefs and acceptance of sexuality, transgender men and the occurrence of sexual abuse occurring within a family. The men also talked about how the perpetrator hindered them reaching out for support, this included threats from the perpetrator and the perpetrator shifting blame onto the men.

“The reason it took me so long to go forward to them was I was deeply in debt and I needed to pay off this debt before I did anything or could go anywhere.” [Participant 5]

“Q: At this time, did you ever think about reporting, seeking help from any sort of support service? A: No. I was living under her roof, she gave me alcohol and I just had to deal with whatever came out of that.” [Participant 6]

“I knew as well, or at least there was a part of me that knew that I was gay or at least interested in men, so there was that compounding of being outed and having that connection of other people knowing that or at least suspecting that, and especially being in that religious community, that could have cost me my family, and it did.” [Participant 21]

“I told my Nana the next day and once she called me a liar, I didn’t want to tell anyone ever again. I told the person that was closest to me and they called me a liar so if I told someone that’s not close to me they’ll probably think the same so I’m not going to tell anyone.” [Participant 36]

“You got told growing up while it was happening that if you told anyone your life would be ruined.” [Participant 36]

“Well he [the perpetrator] made me feel like it was my fault.” [Participant 11]

## Discussion

Seeking support, intervention and treatment have been demonstrated to be crucial in mitigating the long-term effects of sexual harm (Burch & Zepeda, 2023; Oosterbaan et al., 2019). Delayed disclosure and inadequate support may exacerbate detrimental outcomes for survivors (Burch & Zepeda, 2023). In the present study, the process of seeking support emerged as a complex and multifaceted journey for the young university men interviewed, characterized by ongoing personal inner conflict, temporal considerations, emotional relief upon disclosure, contextualization of their experiences, and recognition of the ongoing nature of the healing process (Manay & Collin-Vézina, 2021; Wingender & Olesen, 2023). Across the interviews, the men described a range of services and people that they had reached out to. Supports sought were talked about as multifaceted and included family, friends, formal support services such as healthcare providers, and legal avenues such as the Police (Patterson et al., 2023; Wingender & Olesen, 2023). The men described that they had reached out for support at various time points during and/or after their sexual harm experiences, and that interaction with formal support services often came about via presentations not directly related to their experience of sexual harm (Dixon et al., 2023; Wingender & Olesen, 2023). Additionally, men reported that the act of telling someone signaled the beginning of an ongoing process and some described experiencing secondary victimization where the experience of seeking support was traumatic. The men also talked about the disempowerment and re-traumatization they experienced throughout the legal process (Jackson et al., 2017; Thomas & Kopel, 2023). The interviews conducted shed light on the diverse support needs of the young men who had experienced sexual harm, highlighting three prominent themes.

Firstly, there is a clear need for increased awareness of sexual harm experienced by men (Carlisle & Schmitz, 2023; Wingender & Olesen, 2023). This should include information about what sexual harm looks like for men (Carlisle & Schmitz, 2022; Patterson et al., 2022), recognition of its manifestations, and assurance that others have undergone similar experiences. These findings underscore the importance of dispelling myths and providing societal education to help men reframe their abuse experiences and realize they are not alone. A heightened awareness of sexual harm experienced by men may help encourage men to seek support for their experiences of sexual harm and may also better prepare those more informal support givers (such as friends and family) and clinical healthcare providers with the tools to support those men who have experienced sexual harm.

Secondly, the young men interviewed articulated a need for clarity around formal support services (such as walk-in clinics and telephone helplines), including what services are available, what they entail, and whether they are inclusive of male survivors (see also Pijlman et al., 2023). This points to the necessity for awareness, transparency, and outreach efforts from support organizations to alleviate hesitancy among men who have experienced sexual harm to seek assistance.

Lastly, the interviews underscored the need for a diverse range of support modalities to address the multifaceted needs of men who had experienced sexual harm, encompassing practical support, therapeutic interventions, and advocacy services (Patterson et al., 2023). This highlights the importance of tailoring support options to individual needs and preferences (Dixon et al., 2023; Pijlman et al., 2023).

Throughout each interview, the young men talked about a variety of barriers that hindered them from seeking support or help (Easton et al., 2014; Machado et al., 2016; Pijlman et al., 2023; Rapsey et al., 2020; Weare et al., 2024). These barriers included difficulties in recognizing their experiences as sexually harmful, a reluctance to acknowledge a need for support, preconceived notions about what seeking help might be like, a range of emotional barriers including shame embarrassment and fear of not being believed, lack of consideration of previous (or history of) sexual abuse experiences, and limited accessibility to services.

In addition to these individual-level barriers, a number of overarching social and contextual influences further complicated the support-seeking process for these men (Easton et al., 2014; Machado et al., 2016). The young men discussed how factors such as living situations, cultural norms, religious beliefs, family dynamics, and perpetrator factors interacted with and exacerbated existing individual-level barriers. For instance, financial constraints and debt tied individuals to abusive environments, while cultural and religious influences reinforced feelings of shame and suppressed disclosure. Moreover, threats and blame-shifting tactics employed by perpetrators further deterred survivors from seeking assistance. Addressing these barriers may help increase the number of men seeking support for their experiences of sexual harm.

The findings of this study need to be considered in light of its limitations. The interviews were from a self-selected group of university students from specific regions of NZ. To broaden the understanding of men’s experiences of seeking support the experiences of a more diverse range of men, such as non-university men and older men, need to be included. The results reported here do however provide important insights that are relevant to the provision of sexual harm support services to men, especially those for university students, in NZ. This supports past research demonstrating the need for specific sexual violence prevention efforts for students (Graham et al., 2021; Mushonga et al., 2021). We have focused on the barriers encountered by men as they sought support. Future studies could also examine the enablers that encourage men to seek support, which often mirror barriers but can also provide specific solutions (Dixon et al., 2023). Here we have taken an inclusive definition of “support seeking” that includes seeking informal support from avenues such as friends and family, seeking clinical help such as psychological support from healthcare professionals, and disclosure to legal services such as the Police. Future research needs to tease these apart and examine the barriers and enablers to support seeking from each of these formal and informal avenues.

The interviews with the young men in this study suggest that formal support services need to consider gender-inclusive and gender-sensitive processes beyond services for female survivors of sexual harm such as accessibility, marketing, and clinical practices (Dixon et al., 2023; Lowe, 2018; O’Gorman et al., 2024; Wingender & Olesen, 2023). Moreover, not all those who have experienced sexual harm reach out to formal support services so there is a need to consider how to support and inform the potential supporters (e.g., family and friends) of those who have experienced sexual harm (Liddle et al., 2021; Patterson et al., 2023). In addition, these findings underscore the need for a holistic approach to supporting male survivors of sexual harm (Bach et al., 2021; Dixon et al., 2023) that addresses gender norms, myths about male survivors, acknowledges the diversity among male survivors and their complex lived experiences, and that addresses both individual-level barriers and broader systemic factors, to create an environment conducive to support seeking for men.

## References

[bibr1-08862605241308297] Allen C. T. Ridgeway R. Swan S. C. (2015). College students’ beliefs regarding help seeking for male and female sexual assault survivors: Even less support for male survivors. Journal of Aggression, Maltreatment & Trauma, 24(1), 102–115. 10.1080/10926771.2015.982237

[bibr2-08862605241308297] Andersen T. H. (2013). Against the wind: Male victimization and the ideal of manliness. Journal of Social Work, 13(3), 231–247. 10.1177/1468017311410002

[bibr3-08862605241308297] Artime T. M. McCallum E. B. Peterson Z. D. (2014). Men’s acknowledgment of their sexual victimization experiences. Psychology of Men & Masculinity, 15(3), 313. 10.1037/a0033376

[bibr4-08862605241308297] Bach M. H. Beck Hansen N. Ahrens C. Nielsen C. R. Walshe C. Hansen M. (2021). Underserved survivors of sexual assault: A systematic scoping review. European Journal of Psychotraumatology, 12(1), 1895516. 10.1080/20008198.2021.1895516 33889311 PMC8043556

[bibr5-08862605241308297] Barth J. Bermetz L. Heim E. Trelle S. Tonia T. (2013). The current prevalence of child sexual abuse worldwide: A systematic review and meta-analysis. International journal of public health, 58, 469–483. 10.1007/s00038-012-0426-1 23178922

[bibr6-08862605241308297] Beres M. A. Stojanov Z. Graham K. Treharne G. J. (2020). Sexual assault experiences of university students and disclosure to health professionals and others. New Zealand Medical Journal, 133(1523), 55–64. 33032303

[bibr7-08862605241308297] Braun V. Clarke V. (2022). Thematic analysis: A practical guide. SAGE Publications Ltd.

[bibr8-08862605241308297] Burch E. Zepeda S. (2023). Survivor experiences of male childhood sexual abuse: A literature review. Psychology from the Margins, 5(1), 4.

[bibr9-08862605241308297] Burton D. L. Miller D. L. Shill C. T. (2002). A social learning theory comparison of the sexual victimization of adolescent sexual offenders and nonsexual offending male delinquents. Child Abuse & Neglect, 26(9), 893–907. 10.1016/s0145-2134(02)00360-5 12433134

[bibr10-08862605241308297] Campbell R. Raja S. (1999). Secondary victimization of rape victims: Insights from mental health professionals who treat survivors of violence. Violence and Victims, 14(3), 261. 10606433

[bibr11-08862605241308297] Carlisle Z. T. Schmitz R. M. (2022). Hidden in plain sight: Men’s lived experiences with sexual violence as college students. The Journal of Men’s Studies, 30(2), 193–212. 10.1177/10608265211050679

[bibr12-08862605241308297] Carlisle Z. T. Schmitz R. M. (2023). ‘I am a man. How could I possibly have been raped? Men making sense of their experiences with sexual violence. Journal of Interpersonal Violence, 38(19–20), 10514–10541. 10.1177/08862605231174500 37222535

[bibr13-08862605241308297] DeJong C. Morgan S. J. Cox A. (2020). Male rape in context: Measures of intolerance and support for male rape myths (MRMs). Criminal Justice Studies, 33(3), 195–212. 10.1080/1478601X.2020.1786278

[bibr14-08862605241308297] Depraetere J. Vandeviver C. Beken T. V. Keygnaert I. (2020). Big boys don’t cry: A critical interpretive synthesis of male sexual victimization. Trauma, Violence, & Abuse, 21(5), 991–1010. 10.1177/1524838018816979 PMC7444022 30554559

[bibr15-08862605241308297] Dixon L. Treharne G. Pettie M. Bowden C. Patterson T. Beres M. Mirfin-Veitch B. Shaw R. Eketone-Kelly A. Ashdown J. (2023). Male survivors of sexual violence and abuse (SVA): Barriers and facilitators to reporting and accessing services. Ministry of Social Development.

[bibr16-08862605241308297] Dixon L. Treharne G. J. Celi E. M. Hines D. A. Lysova A. V. Douglas E. M. (2022). Examining men’s experiences of abuse from a female intimate partner in four English-speaking countries. Journal of Interpersonal Violence, 37(3–4), 1311–1337. 10.1177/0886260520922342 32468917

[bibr17-08862605241308297] Easton S. D. (2012). The disclosure process for men with histories of sexual abuse. Clinical Social Work Journal, 12, 1–12. 10.1007/s10615-012-0420-3

[bibr18-08862605241308297] Easton S. D. Renner L. M. O’Leary P. (2013). Suicide attempts among men with histories of child sexual abuse: Examining abuse severity, mental health, and masculine norms. Child Abuse & Neglect, 37(6), 380–387. 10.1016/j.chiabu.2012.11.007 23313078

[bibr19-08862605241308297] Easton S. D. Saltzman L. Y. Willis D. G. (2014). “Would you tell under circumstances like that?”: Barriers to disclosure of child sexual abuse for men. Psychology of Men & Masculinity, 15(4), 460–469. https:// 10.1037/a0034223

[bibr20-08862605241308297] Eisenberg M. E. Lust K. Mathiason M. A. Porta C. M. (2021). Sexual assault, sexual orientation, and reporting among college students. Journal of Interpersonal Violence, 36(1–2), 62–82. 10.1177/0886260517726414 29294876

[bibr21-08862605241308297] Gallo-Silver L. Anderson C. M. Romo J. (2014). Best clinical practices for male adult survivors of childhood sexual abuse: “do no harm”. The Permanente Journal, 18(3), 82. 10.7812/TPP/14-009 25106042 PMC4116270

[bibr22-08862605241308297] Gibson K. Morgan M. (2013). Narrative research on child sexual abuse: Addressing perennial problems in quantitative research. Qualitative Research in Psychology, 10(3), 298–317. 10.1080/14780887.2011.606597

[bibr23-08862605241308297] Graham K. Potterton H. Mihaere T. Carrington B. Treharne G. J. Beres M. A. (2021). Balancing community input and established research: Findings from the development of a sexual violence prevention campaign. Journal of School Violence, 20(3), 288–300. 10.1080/15388220.2021.1897017

[bibr24-08862605241308297] Hammond L. Ioannou M. Fewster M. (2017). Perceptions of male rape and sexual assault in a male sample from the United Kingdom: Barriers to reporting and the impacts of victimization. Journal of Investigative Psychology and Offender Profiling, 14(2), 133–149. 10.1002/jip.1462

[bibr25-08862605241308297] Jackson M. A. Valentine S. E. Woodward E. N. Pantalone D. W. (2017). Secondary victimization of sexual minority men following disclosure of sexual assault: “Victimizing me all over again. . .”. Sexuality Research and Social Policy, 14, 275–288.

[bibr26-08862605241308297] Jouriles E. N. Nguyen J. Krauss A. Stokes S. L. McDonald R. (2022). Prevalence of sexual victimization among female and male college students: A methodological note with data. Journal of Interpersonal Violence, 37(11–12), NP8767–NP8792. 10.1177/0886260520978198 33300396

[bibr27-08862605241308297] Kia-Keating M. Grossman F. K. Sorsoli L. Epstein M. (2005). Containing and resisting masculinity: narratives of renegotiation among resilient male survivors of childhood sexual abuse. Psychology of Men & Masculinity, 6(3), 169. 10.1037/1524-9220.6.3.169

[bibr28-08862605241308297] Larkin M. Watts S. Clifton E. (2006). Giving voice and making sense in interpretative phenomenological analysis. Qualitative Research in Psychology, 3(2), 102–120. 10.1191/1478088706qp062oa

[bibr29-08862605241308297] Liddle S. K. Robinson L. Vella S. A. Deane F. P. (2021). Profiles of mental health help seeking among Australian adolescent males. Journal of Adolescence, 92, 34–45. 10.1016/j.adolescence.2021.08.010 34416479

[bibr30-08862605241308297] Lowe M. (2018). Male sexual assault survivors: Lessons for UK services. Journal of Aggression, Conflict and Peace Research, 10(3), 181–188.

[bibr31-08862605241308297] Lowe M. Balfour B. (2015). The unheard victims. The Psychologist, 28, 118–121.

[bibr32-08862605241308297] Luetke M. Giroux S. Herbenick D. Ludema C. Rosenberg M. (2021). High prevalence of sexual assault victimization experiences among university fraternity men. Journal of Interpersonal Violence, 36(23–24), 11755–11767. 10.1177/0886260519900282 31984858

[bibr33-08862605241308297] Machado A. Hines D. Matos M. (2016). Help-seeking and needs of male victims of intimate partner violence in Portugal. Psychology of Men & Masculinity, 17(3), 255. 10.1037/men0000013

[bibr34-08862605241308297] Manay N. Collin-Vézina D. (2021). Recipients of children’s and adolescents’ disclosures of childhood sexual abuse: A systematic review. Child Abuse & Neglect, 116, 104192. 10.1016/j.chiabu.2019.104192 31564382

[bibr35-08862605241308297] Maniglio R. (2009). The impact of child sexual abuse on health: A systematic review of reviews. Clinical Psychology Review, 29(7), 647–657. 10.1016/j.cpr.2009.08.003 19733950

[bibr36-08862605241308297] Mejia X. E. (2005). Gender matters: Working with adult male survivors of trauma. Journal of Counseling & Development, 83(1), 29–40. 10.1002/j.1556-6678.2005.tb00577.x

[bibr37-08862605241308297] Ministry of Justice. (2020). New Zealand Crime and Victims Survey. Key findings Cycle 3. Sexual violence and violence by family members [Data file]. Ministry of Justice.

[bibr38-08862605241308297] Monk-Turner E. Light D. (2010). Male sexual assault and rape: who seeks counseling? Sexual Abuse, 22(3), 255–265. 10.1177/1079063210366271 20713746

[bibr39-08862605241308297] Murphy-Oikonen J. Egan R. (2022). Sexual and gender minorities: Reporting sexual assault to the police. Journal of Homosexuality, 69(5), 773–795. 10.1080/00918369.2021.1892402 33722182

[bibr40-08862605241308297] Mushonga D. R. Fedina L. Bessaha M. L. (2021). College student perceptions of institutional responses to sexual assault reporting and general help-seeking intentions. Journal of American College Health, 69(6), 585–591. 10.1080/07448481.2019.1705827 31995447

[bibr41-08862605241308297] Nicholas A. Krysinska K. King K. E. (2022). A rapid review to determine the suicide risk and risk factors of men who are survivors of sexual assault. Psychiatry Research, 317, 114847–114849. 10.1016/j.psychres.2022.114847 36126347

[bibr42-08862605241308297] O’Gorman K. Pilkington V. Seidler Z. Oliffe J. L. Peters W. Bendall S. Rice S. M. (2024). Childhood sexual abuse in boys and men: The case for gender-sensitive interventions. Psychological Trauma: Theory, Research, Practice, and Policy, 16(Suppl 1), S181–S189. 10.1037/tra0001520 37326539

[bibr43-08862605241308297] O’Leary P. Easton S. D. Gould N. (2017). The effect of child sexual abuse on men: Toward a male sensitive measure. Journal of Interpersonal Violence, 32(3), 423–445. 10.1177/0886260515586362 26033613

[bibr44-08862605241308297] Oosterbaan V. Covers M. L. Bicanic I. A. Huntjens R. J. de Jongh A. (2019). Do early interventions prevent PTSD? A systematic review and meta-analysis of the safety and efficacy of early interventions after sexual assault. European Journal of Psychotraumatology, 10(1), 1682932. 10.1080/20008198.2019.1682932 31762949 PMC6853210

[bibr45-08862605241308297] Patterson T. Campbell A. La Rooy D. Hobbs L. Clearwater K. Rapsey C. (2023). Impact, ramifications and taking back control: A qualitative study of male survivors of childhood sexual abuse. Journal of Interpersonal Violence, 38(1–2), 1868–1892. 10.1177/08862605221094629 35487882

[bibr46-08862605241308297] Patterson T. Hobbs L. Treharne G. J. Beres M. (2022). Seeking of help and support after experiencing sexual harm: Considerations for cisgender women, cisgender men and gender-diverse people. The New Zealand Medical Journal, 135(1562), 56–62. 10.26635/6965.5686 36137767

[bibr47-08862605241308297] Peterson Z. D. Voller E. K. Polusny M. A. Murdoch M. (2011). Prevalence and consequences of adult sexual assault of men: Review of empirical findings and state of the literature. Clinical Psychology Review, 31(1), 1–24. 10.1016/j.cpr.2010.08.006 21130933

[bibr48-08862605241308297] Petersson C. C. Plantin L. (2019). Breaking with norms of masculinity: Men making sense of their experience of sexual assault. Clinical Social Work Journal, 47(4), 372–383. 10.1007/s10615-019-00699-y

[bibr49-08862605241308297] Pijlman V. Eichelsheim V. Pemberton A. de Waardt M. (2023). “Sometimes it seems easier to push it away”: A study into the barriers to help-seeking for victims of sexual violence. Journal of Interpersonal Violence, 38(11–12), 7530–7555. 10.1177/08862605221147064 36710513

[bibr50-08862605241308297] Rapsey C. Campbell A. Clearwater K. Patterson T. (2020). Listening to the therapeutic needs of male survivors of childhood sexual abuse. Journal of Interpersonal Violence, 35(9–10), 2033–2054. 10.1177/0886260517701453 29294699

[bibr51-08862605241308297] Rice S. M. Easton S. D. Seidler Z. E. Oliffe J. L. (2022). Sexual abuse and mental ill health in boys and men: what we do and don’t know. BJPsych Open, 8(4), e110. 10.1192/bjo.2022.508 PMC9230611 35678473

[bibr52-08862605241308297] Salter D. McMillan D. Richards M. Talbot T. Hodges J. Bentovim A. Hastings R. Stevenson J. Skuse D. (2003). Development of sexually abusive behaviour in sexually victimised males: A longitudinal study. The Lancet, 361(9356), 471–476. 10.1016/S0140-6736(03)12466-X 12583946

[bibr53-08862605241308297] Smith J. A. Osborn M. (2015). Interpretative phenomenological analysis. In Smith J. A. (Ed.), Qualitative psychology: A practical guide to research methods (pp. 53–80). SAGE.

[bibr54-08862605241308297] Thomas J. C. Kopel J. (2023). Male victims of sexual assault: A review of the literature. Behavioral Sciences, 13(4), 304. 37102818 10.3390/bs13040304 PMC10135558

[bibr55-08862605241308297] Turchik J. A. Edwards K. M. (2012). Myths about male rape: A literature review. Psychology of Men & Masculinity, 13(2), 211. 10.1037/a0023207

[bibr56-08862605241308297] Weare S. Hulley J. Craig D. (2024). ‘Nobody believes you if you’re a bloke’: Barriers to disclosure and help-seeking for male forced-to-penetrate victims/survivors. International Review of Victimology, 30(3), 596–611. 10.1177/02697580241238768

[bibr57-08862605241308297] Widanaralalage B. K. Hine B. A. Murphy A. D. Murji K. (2022). “I didn’t feel I was a victim”: A phenomenological analysis of the experiences of male-on-male survivors of rape and sexual abuse. Victims & Offenders, 17(8), 1147–1172. 10.1080/15564886.2022.2069898

[bibr58-08862605241308297] Willis D. G. Zucchero T. L. DeSanto-Madeya S. Ross R. Leone D. Kaubris S. Moll K. Kuhlow E. Easton S. D. (2014). Dwelling in suffering: Barriers to men’s healing from childhood maltreatment. Issues in Mental Health Nursing, 35(8), 569–579. 10.3109/01612840.2013.856972 25072209

[bibr59-08862605241308297] Wingender A. M. Olesen M. L. (2023). Male victims’ acknowledgement of sexual assault and their help-seeking process. A qualitative study. The Journal of Men’s Studies, 32(2), 325–345. 10.1177/10608265231215078

[bibr60-08862605241308297] Zarchev M. Ruijne R. E. Mulder C. L. Kamperman A. M. (2022). Prevalence of adult sexual abuse in men with mental illness: Bayesian meta-analysis. BJPsych Open, 8(1), e16. 10.1192/bjo.2021.1069 PMC8715257 34915966

